# Clinical Outcomes of Monopolar Versus Bipolar Transurethral Resection of the Prostate for Benign Prostatic Enlargement: A Prospective Comparative Study

**DOI:** 10.7759/cureus.115386

**Published:** 2026-08-29

**Authors:** Amar Kumar Repaka, Vyza Vishnu Vardhana Reddy, Arun Kumar V

**Affiliations:** 1 Department of Urology, All India Institute of Medical Sciences (AIIMS), Bibinagar, Bibinagar, IND; 2 Department of Urology, Kamineni Academy of Medical Sciences and Research Centre, Hyderabad, IND

**Keywords:** benign prostatic enlargement, bipolar transurethral resection, lower urinary tract symptoms, monopolar transurethral resection, transurethral resection syndrome

## Abstract

Benign prostatic enlargement (BPE) is a common cause of lower urinary tract symptoms (LUTS) in older men, and transurethral resection of the prostate (TURP) remains an established surgical treatment when conservative management is inadequate. However, uncertainty persists regarding whether bipolar TURP offers meaningful perioperative safety advantages without compromising functional efficacy. This prospective, non-randomized comparative study evaluated monopolar and saline bipolar TURP in 100 patients, including 50 (50%) patients in the monopolar group and 50 (50%) patients in the bipolar group. Preoperative and one-month postoperative assessments included International Prostate Symptom Score (IPSS), quality of life (QoL) score, maximum urinary flow rate, hemoglobin, packed cell volume (PCV), serum sodium, resection time, and early postoperative complications. Both techniques produced substantial and broadly comparable functional improvement. Maximum urinary flow rate increased from 9.76 to 17.74 mL/s after monopolar TURP and from 9.55 to 17.54 mL/s after bipolar TURP. International Prostate Symptom Score decreased by 10.50 and 9.54 points, while quality-of-life score improved by 2.08 and 2.04 points, respectively. Bipolar TURP required a longer mean resection time than monopolar TURP (44.92 versus 41.74 minutes; p=0.001). Postoperative serum sodium declined more markedly after monopolar TURP, and transurethral resection syndrome occurred in 2 (4%) patients in the monopolar group and 0 (0%) patients in the bipolar group. Hemoglobin and packed cell volume reductions were modest in both groups. Overall, bipolar TURP preserved short-term functional efficacy while offering a more favorable electrolyte and fluid-related safety profile.

## Introduction

Benign prostatic hyperplasia (BPH) is a non-malignant pathological process characterized by the proliferation of epithelial and stromal tissue, primarily in the transition zone of the prostate, and it becomes more prevalent with age [1]. Histological BPH can lead to benign prostatic enlargement (BPE), bladder outlet obstruction, and lower urinary tract symptoms (LUTS), which are not synonymous, with prostate size not always being correlated with symptoms or degree of obstruction [1]. Multiple factors play a role in the pathogenesis of BPH, such as androgen-dependent signaling, stromal-epithelial interactions, growth factor activity, inflammation, extracellular matrix remodeling, and altered regulation of cellular proliferation and apoptosis [2]. These mechanisms are responsible for differences in disease expression, disease progression, and treatment response [2].

Transurethral resection of the prostate (TURP) is a well-established surgery that can be performed in men with troublesome LUTS or complications associated with BPE where conservative or pharmacological therapies are not effective [3]. The monopolar TURP technique requires a non-conductive irrigation fluid and an electrical circuit that traverses the patient, while the bipolar technique can be resected in normal saline and allows the focusing of the electrical current in the operative field [3]. There is some evidence that both procedures are effective in aiding urinary symptoms, maximum urinary flow rate, sexual function, and quality of life (QoL), but there may be differences in the perioperative results [3]. Results from a meta-analysis also indicate similar functional efficacy, and bipolar TURP can minimize fluid-related complications, transurethral resection syndrome, catheterization time, and hospital stay [4].

Standardization of symptom assessment is important since the clinical burden of BPH is not only dependent on the anatomical enlargement but also on the severity of symptoms reported by the patient [5]. The American Urological Association symptom index was later combined into the International Prostate Symptom Score (IPSS), and it is a valid method to measure the symptoms of the prostate and their severity before and after treatment [5]. In addition to symptom scoring, assessment of quality of life (QoL) measures the bother and functional impairment experienced by the patient with LUTS [6]. This is significant as men with similar symptom scores might have different levels of dissatisfaction and treatment preference [6]. Prostate size and urinary flow rate, post-void residual (PVR) urine, and other anatomical or physiological parameters are also imperfectly correlated with the severity of symptoms [7]. This means that a score of symptoms, quality of life, uroflowmetry, laboratory changes, and complications after the surgery should be measured when performing a comparative surgical evaluation, not just one single outcome [7].

Similar improvements in IPSS, maximum urinary flow rate, and quality of life have been reported after monopolar and bipolar TURP, and the risk of dilutional hyponatremia and transurethral resection syndrome is reduced after bipolar resection [8]. Medical therapy may include 5-alpha-reductase inhibition, which can shrink the prostate and resolve symptoms in some patients, but it has a slow onset of effect and may not be enough in severe obstruction or complicated disease [9,10]. It is important to remember that LUTS should be interpreted in a broad context as symptoms of bladder dysfunction, ageing, neurological disease, and non-prostatic causes can all contribute to LUTS.

Recent comparative studies further support similar functional results between the two techniques, and there are potential differences in blood loss, electrolyte disturbance, catheterization, and recovery [10,11]. The differences in the design, sample size, prostate volume, equipment, operator experience, and postoperative protocols, however, may account for the variations found between different institutions [12]. There is also a lack of regional evidence due to brief follow-up periods and inconsistent reporting of symptoms, flow, and biochemical and complication outcomes [13]. During transurethral surgery, the resectoscope was improved in terms of visualization, irrigation, suction, and pressure control within the bladder [14]. The evolution of endoscopic prostate surgery has been closely linked to progressive refinements in resectoscope design and electrosurgical techniques, which established the technical foundation for modern transurethral resection procedures [15]. However, variability persists in the reported short-term perioperative outcomes between monopolar and bipolar TURP techniques across different studies. Thus, there is a need for future, institution-based studies that evaluate symptom improvement, urinary flow, quality of life, resection time, hematological and electrolyte changes, and early postoperative complications all in one.

Objectives of the study

The primary objective of this study was to compare the short-term functional outcomes of conventional monopolar TURP and saline bipolar TURP in patients with benign prostatic enlargement, assessed using the International Prostate Symptom Score (IPSS), quality of life (QoL) score, and maximum urinary flow rate (Qmax). The secondary objective was to compare perioperative and early postoperative outcomes, including resection time, hemoglobin, packed cell volume (PCV), serum sodium changes, and early postoperative complications. Post-void residual urine volume was not included as an outcome in the present study.

## Materials and methods

Study design and setting

Between July 2021 and January 2023, the Department of Urology at Kamineni Academy of Medical Sciences and Research Centre in Hyderabad, India, conducted a non-randomized prospective comparative study. No formal blinding was employed because the operative technique and equipment differed between monopolar and bipolar TURP and were therefore identifiable to the treating surgical team. In patients with benign prostatic enlargement, the study compared the clinical and early postoperative outcomes of saline bipolar TURP with conventional monopolar TURP. The study sample comprised 100 eligible patients, with 50 patients in each treatment group, based on the planned recruitment during the study period.

Study population

Patients fulfilling the eligibility criteria were allocated to either conventional monopolar TURP or saline bipolar TURP in a non-randomized manner. The choice of surgical technique was based on the treating surgeon's clinical judgment and availability of the respective electrosurgical system at the time of surgery. Group 1 comprised 50 patients who underwent conventional monopolar TURP, and group 2 comprised 50 patients who underwent saline bipolar TURP. Because allocation was non-randomized, the possibility of selection bias and residual differences between the treatment groups cannot be excluded.

Inclusion criteria

The patients were included if they were >50 years old, had LUTS secondary to BPE, had a prostate size of >30 g by ultrasonography of the kidneys, ureters, and bladder, and had a maximum urinary flow rate of <15 mL/s. The upper limit for prostate volume was not defined.

Exclusion criteria

Those with prostate cancer, urethral stricture, neurogenic bladder, prostatitis, active urinary tract infection (UTI), previous prostate surgery, coagulopathy, vesical calculi, or bladder carcinoma were excluded.

Ethical consideration

The study was approved by the Institutional Ethics Committee of Kamineni Academy of Medical Sciences and Research Centre (KAMSRC), Kamineni Hospitals, Hyderabad (approval/reference number: ECR/58/Inst/AP/2013/RR-19). The committee reviewed the study on July 28, 2021, and the approval letter was issued on July 29, 2021. Informed consent was obtained from all participants before their inclusion in the study.

Preoperative evaluation

A complete clinical history was obtained for each subject. The quality of life score and preoperative IPSS were measured. Comorbid conditions such as ischemic heart disease, diabetes mellitus, and hypertension were noted. Digital rectal and genital examinations were part of the general and systemic checkup.

Complete urine examination, urine culture and sensitivity, complete hemogram, renal function tests, serum sodium and potassium, coagulation parameters, blood grouping, and typing were among the other tests performed before surgery. Patients with positive urine cultures received the appropriate antibiotics. Kidney, ureter, and bladder ultrasonography and plain radiography were performed. Suitable patients underwent uroflowmeter testing. Before surgery, anesthetic fitness was assessed.

Surgical procedure

The surgery was performed under general anesthetic and in the lithotomy position. The initial cystourethroscopy was done to assess the anterior urethra, verumontanum, prostate gland, bladder mucosa, and both the ureteric orifices. The conventional monopolar TURP was carried out using a Karl Storz 24 Fr non-continuous-flow resectoscope and using irrigating fluid of glycine and the active working element of the Baum Rucker. Bipolar TURP was performed using a bipolar system with normal saline as the irrigating fluid. The bipolar system was used with normal saline as the irrigating fluid for saline bipolar TURP. Experienced senior professors carried out all the resections.

The resection time was considered as the time interval from the start of resection to the removal of the resectoscope sheath. Complications were reported during the operation. A urinary catheter (22-Fr three-way) was inserted, and continuous bladder irrigation started at the end of the procedure. The tissue removed by surgery was submitted for pathological examination.

Postoperative management

Hematuria, altered sensorium, and changes in vital parameters were monitored in patients following surgery. Hemoglobin, packed cell volume, serum sodium, and serum potassium levels were measured using blood samples. Up until the following day, the bladder was routinely irrigated, with further irrigation administered as needed. On the fourth day following surgery, the patients were discharged, and the urinary catheter was removed.

Follow-up and outcome assessment

Patients were evaluated at one-month follow-up. Histopathology assessment and International Prostate Symptom Score and quality of life scores were performed post-surgery. Uroflowmetry was also performed at follow-up. Preoperative and postoperative assessments were made of International Prostate Symptom Score, quality of life score, maximum urinary flow rate, level of hemoglobin, packed cell volume, and serum sodium. IPSS and QoL scores were used as standardized patient-reported measures, while uroflowmetry was used for objective assessment of Qmax. Early postoperative complications were identified during the postoperative hospital course and at follow-up and were recorded according to their clinical presentation. Clot retention requiring evacuation, transurethral resection syndrome, and failure to void following catheter removal were specifically recorded. Improvement of symptoms and postoperative complications were compared between the conventional monopolar TURP and saline bipolar TURP groups.

Statistical analysis

Descriptive statistics were used to describe the study population. Continuous variables were expressed as mean ± standard deviation (SD), while categorical variables were presented as frequencies and percentages. Between-group comparisons of continuous variables were performed using the Z-test. Preoperative and postoperative values were summarized separately, and Qmax analyses were based on the available measurements. Early postoperative complications were reported descriptively. Statistical significance was based on two-sided p-values, with p<0.05 considered statistically significant. Statistical analyses were performed using SPSS version 17 (SPSS Inc., Chicago, Ill) and Python (Python Software Foundation, Wilmington, DE).

## Results

Participant characteristics

A total of 100 (100%) male patients with benign prostatic enlargement were included in the study. Of these, 50 (50%) patients underwent conventional monopolar TURP, and 50 (50%) patients underwent saline bipolar TURP. All 100 patients completed the one-month follow-up; therefore, there was no loss to follow-up, and no patient was excluded from the final analysis. The ages of both groups ranged from 50 to 80 years. At presentation, indwelling urinary catheters were present in 8 (16%) patients in the monopolar group and 7 (14%) patients in the bipolar group. Preoperative catheterization in these patients was required for urinary retention associated with bladder outlet obstruction secondary to benign prostatic enlargement. Diabetes mellitus was present in 9 (18%) patients in the monopolar group and 8 (16%) patients in the bipolar group. Hypertension was present in 8 (16%) patients in each group. The clinical and general demographic characteristics of the two groups were comparable (Table 1).

**Table 1:** Baseline demographic and clinical characteristics TURP: transurethral resection of the prostate, n: number of patients, %: percentage

Characteristic	Monopolar TURP (n=50)	Bipolar TURP (n=50)
Age range, years	50-80	50-80
Indwelling urinary catheter, n (%)	8 (16%)	7 (14%)
Diabetes mellitus, n (%)	9 (18%)	8 (16%)
Hypertension, n (%)	8 (16%)	8 (16%)

The mean age was 64.33±7.10 years in the monopolar group and 65.97±8.27 years in the bipolar group. The mean prostate volume was 35.51±6.14 g in the monopolar group and 35.59±5.78 g in the bipolar group. These findings indicated that the groups were broadly comparable in age and prostate volume. Table 2 compares the mean age and prostate volume between the treatment groups.

**Table 2:** Comparison of age and prostate volume between the study groups TURP: transurethral resection of the prostate, n: number of patients, SD: standard deviation, g: grams

Variable	Monopolar TURP (n=50), mean±SD	Bipolar TURP (n=50), mean±SD
Age, years	64.33±7.10	65.97±8.27
Prostate volume, g	35.51±6.14	35.59±5.78

Preoperative clinical and laboratory parameters

Preoperative maximum urinary flow rate (Qmax) measurements were available for 44 (88%) patients in the monopolar group and 42 (84%) patients in the bipolar group. The mean Qmax was 9.76±0.98 mL/s in the monopolar group and 9.55±1.19 mL/s in the bipolar group. The mean International Prostate Symptom Score (IPSS) was 23.24±2.10 and 22.46±1.76, respectively. The baseline IPSS was significantly higher in the monopolar group than in the bipolar group (p=0.044), indicating a small baseline difference in symptom severity between the groups. This baseline imbalance was considered when interpreting postoperative symptom outcomes.

The mean preoperative hemoglobin was 12.39±0.94 g/dL in the monopolar group and 12.27±1.19 g/dL in the bipolar group. The mean packed cell volume (PCV) was 36.18±2.69% and 35.98±3.08%, respectively. The mean serum sodium was 139.46±3.02 mEq/L in the monopolar group and 139.58±3.16 mEq/L in the bipolar group (Figure 1). The mean quality of life (QoL) scores were 3.90±0.74 and 3.92±0.78, respectively. Apart from IPSS, the reported baseline clinical and laboratory parameters did not differ significantly between the groups. Table 3 summarizes the preoperative clinical and laboratory parameters in both groups.

**Figure 1:**
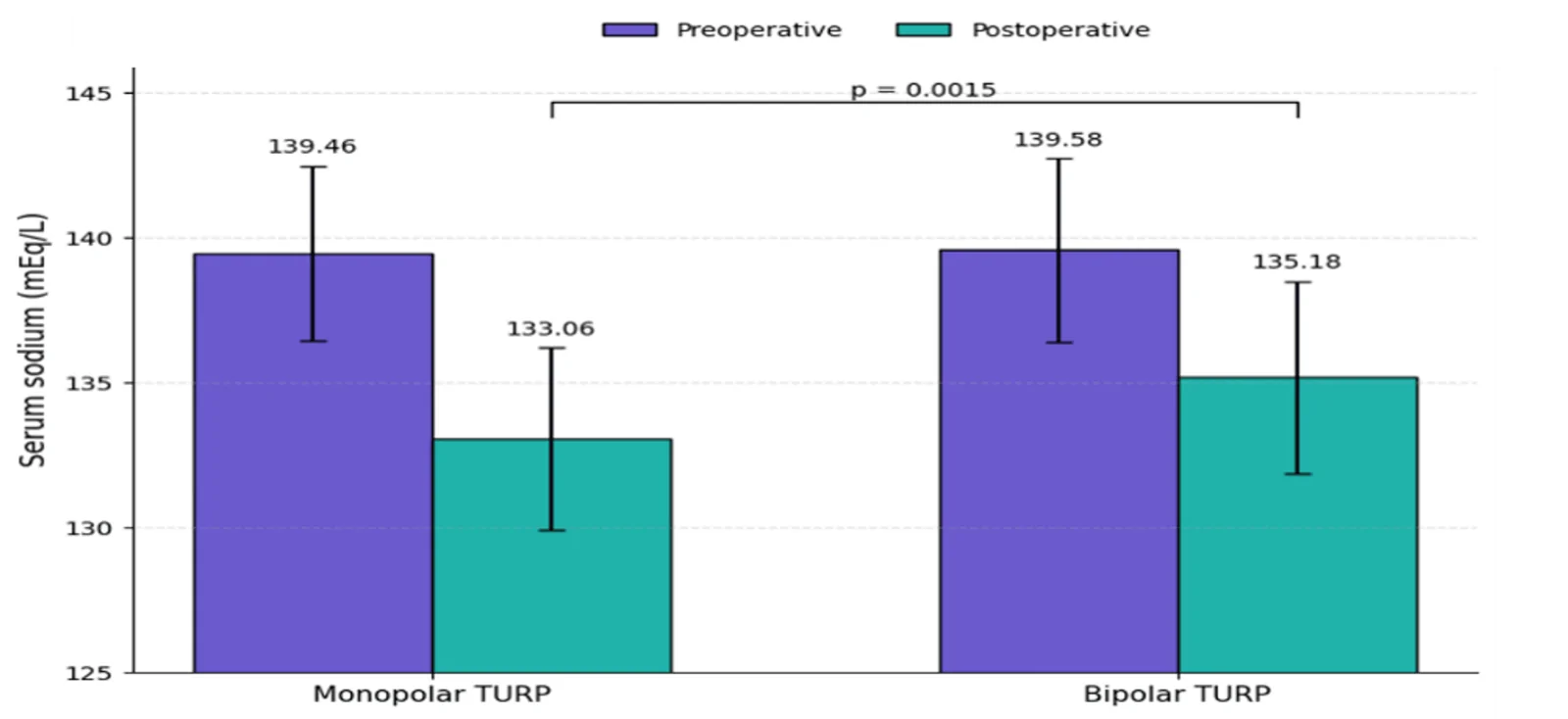
Preoperative and postoperative serum sodium levels TURP: transurethral resection of the prostate

**Table 3:** Preoperative clinical and laboratory parameters TURP: transurethral resection of the prostate, SD: standard deviation, Qmax: maximum urinary flow rate, mL/s: milliliters per second, n: number of patients, IPSS: International Prostate Symptom Score, g/dL: grams per deciliter, PCV: packed cell volume, %: percentage, mEq/L: milliequivalents per liter, QoL: quality of life

Parameter	Monopolar TURP, mean±SD	Bipolar TURP, mean±SD	Reported p-value
Qmax, mL/s	9.76±0.98, n=44 (88%)	9.55±1.19, n=42 (84%)	0.381
IPSS	23.24±2.10	22.46±1.76	0.044
Hemoglobin, g/dL	12.39±0.94	12.27±1.19	0.582
PCV, %	36.18±2.69	35.98±3.08	0.729
Serum sodium, mEq/L	139.46±3.02	139.58±3.16	0.845
QoL score	3.90±0.74	3.92±0.78	0.894

Operative findings

The mean resection time was 41.74±5.14 minutes in the monopolar group and 44.92±4.42 minutes in the bipolar group. The bipolar procedure required a longer resection time, and the reported between-group difference was statistically significant (p=0.001) (Figure 2). The longer resection time observed in the bipolar group may be related to the plasma-field generation mechanism required for bipolar tissue resection. Table 4 demonstrates the significantly longer resection time associated with bipolar TURP.

**Figure 2:**
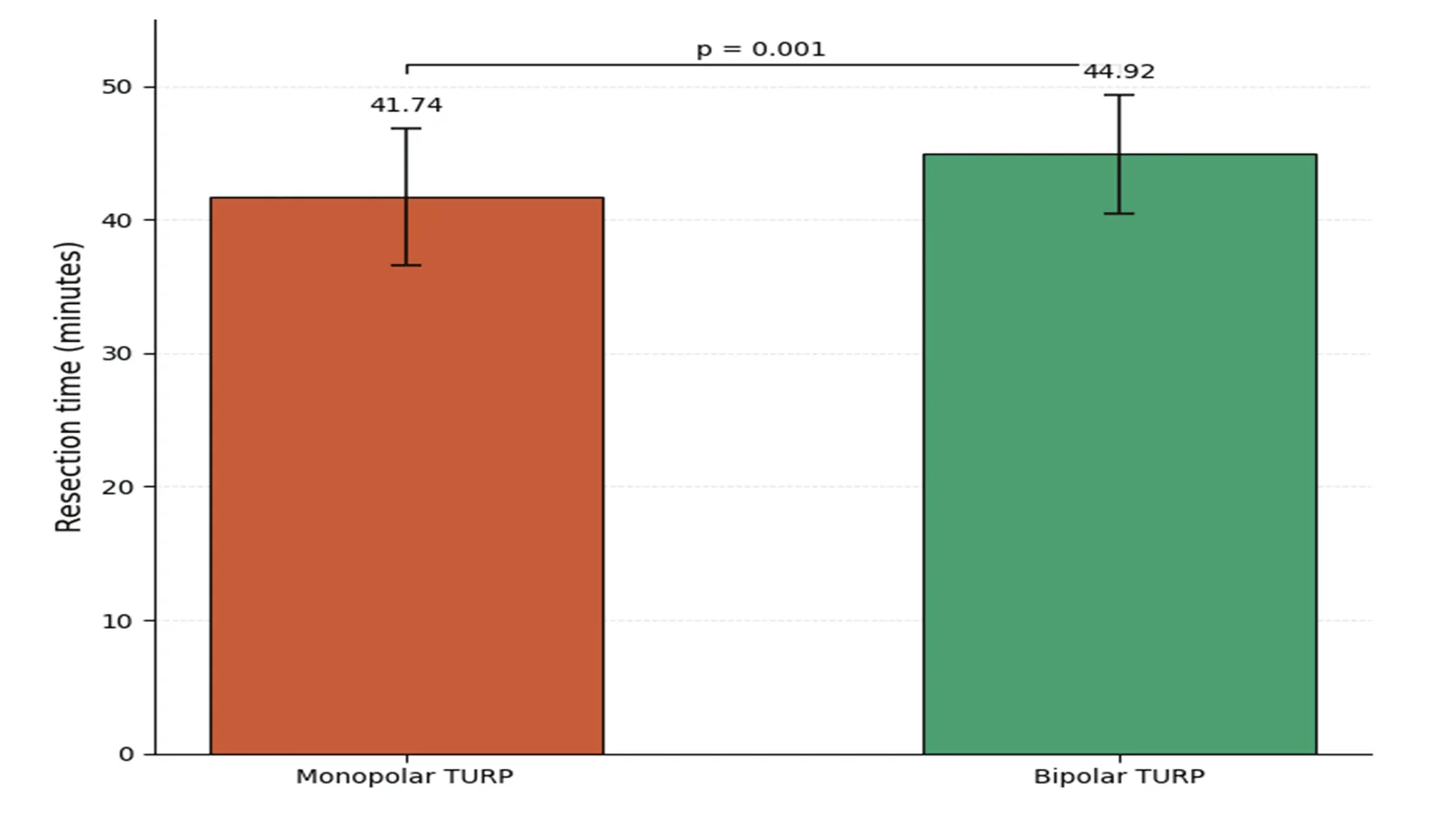
Resection time in the monopolar and bipolar TURP groups TURP: transurethral resection of the prostate

**Table 4:** Comparison of resection time between the study groups TURP: transurethral resection of the prostate, n: number of patients, SD: standard deviation

Variable	Monopolar TURP (n=50), mean±SD	Bipolar TURP (n=50), mean±SD	Reported p-value
Resection time, minutes	41.74±5.14	44.92±4.42	0.001

At the one-month follow-up, the mean Qmax increased to 17.74±0.74 mL/s in the monopolar group and 17.54±0.74 mL/s in the bipolar group. The absolute increases were 7.98 and 7.99 mL/s, respectively. The mean postoperative IPSS decreased to 12.74±1.47 in the monopolar group and 12.92±1.40 in the bipolar group. The corresponding reductions from baseline were 10.50 points in the monopolar group and 9.54 points in the bipolar group. The mean QoL score decreased to 1.82±0.56 and 1.88±0.56, representing improvements of 2.08 and 2.04 points, respectively (Figure 3). Both techniques, therefore, produced substantial and broadly similar improvements in urinary symptoms, urinary flow, and quality of life. Table 5 illustrates the improvement in Qmax, IPSS, and QoL after both procedures.

**Figure 3:**
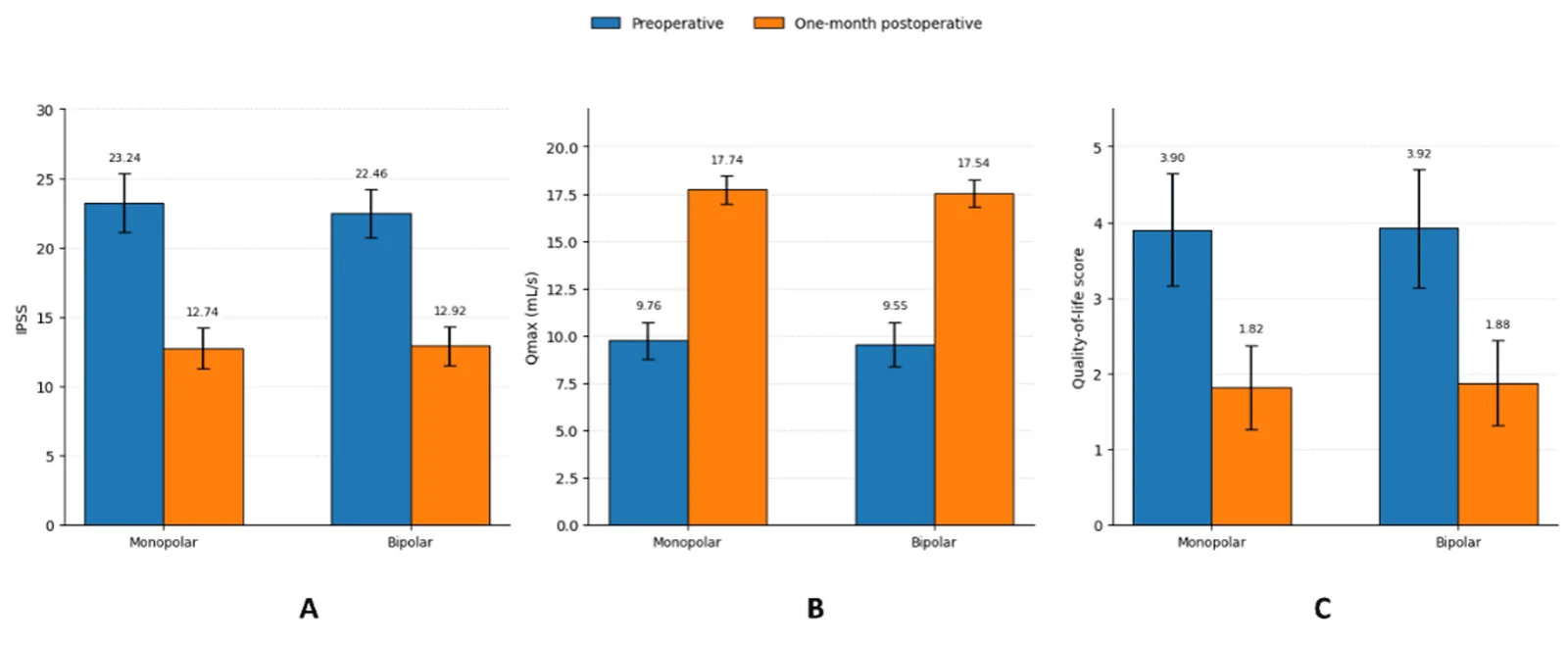
Preoperative and postoperative changes in functional outcomes: (A) IPSS, (B) Qmax, and (C) quality of life IPSS: International Prostate Symptom Score, Qmax: maximum urinary flow rate, QoL: quality of life, mL/s: milliliters per second

**Table 5:** Preoperative and one-month postoperative functional outcomes Qmax: maximum urinary flow rate, mL/s: milliliters per second, IPSS: International Prostate Symptom Score, QoL: quality of life, TURP: transurethral resection of the prostate, SD: standard deviation

Outcome	Group	Preoperative mean±SD	Postoperative mean±SD	Mean change
Qmax, mL/s	Monopolar TURP	9.76±0.98	17.74±0.74	+7.98
	Bipolar TURP	9.55±1.19	17.54±0.74	+7.99
IPSS	Monopolar TURP	23.24±2.10	12.74±1.47	-10.50
	Bipolar TURP	22.46±1.76	12.92±1.40	-9.54
QoL score	Monopolar TURP	3.90±0.74	1.82±0.56	-2.08
	Bipolar TURP	3.92±0.78	1.88±0.56	-2.04

Postoperative hematological and electrolyte changes

The mean postoperative hemoglobin levels in the monopolar and bipolar groups were 11.68±0.93 g/dL and 11.69±1.02 g/dL, respectively. The mean decreases in hemoglobin were 0.71 g/dL after monopolar TURP and 0.58 g/dL after bipolar TURP. The mean postoperative PCV in the monopolar group was 34.44±2.66%, and in the bipolar group, it was 34.12±3.33%. The corresponding decreases were 1.74 and 1.86 percentage points (Figure 4).

**Figure 4:**
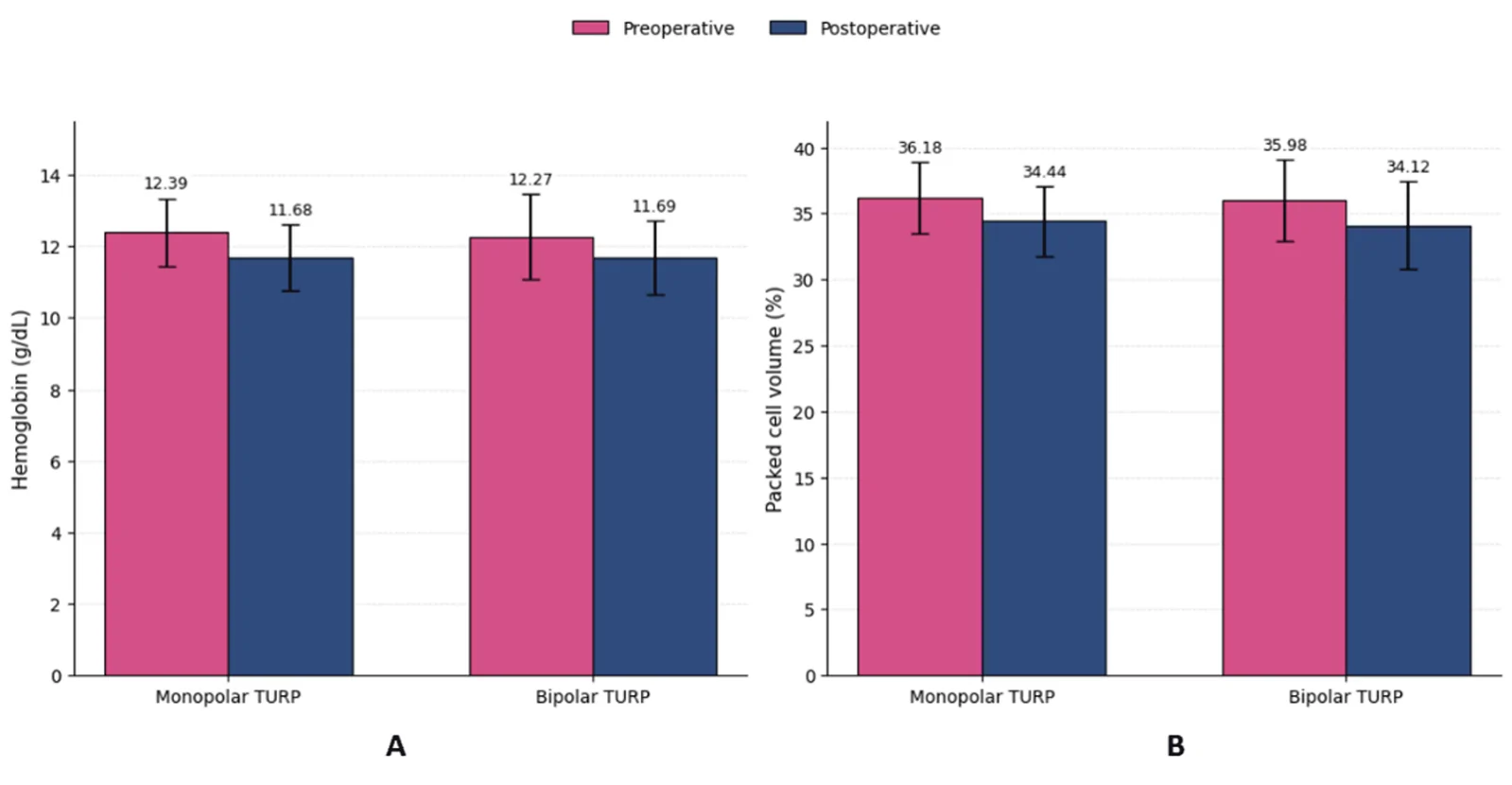
Changes in hematological parameters after TURP: (A) hemoglobin and (B) packed cell volume TURP: transurethral resection of the prostate, g/dL: grams per deciliter, %: percentage

The mean serum sodium decreased from 139.46±3.02 to 133.06±3.16 mEq/L in the monopolar group and from 139.58±3.16 to 135.18±3.32 mEq/L in the bipolar group. The mean Na reductions were 6.40 mEq/L and 4.40 mEq/L, respectively. The serum sodium level after surgery was significantly lower in the monopolar group than in the bipolar group (reported p=0.0015). Table 6 shows the higher postoperative decrease in serum sodium in patients who had monopolar TURP.

**Table 6:** Preoperative and postoperative hematological and electrolyte parameters SD: standard deviation, g/dL: grams per deciliter, TURP: transurethral resection of the prostate, PCV: packed cell volume, %: percentage, mEq/L: milliequivalents per liter

Parameter	Group	Preoperative mean±SD	Postoperative mean±SD	Mean change
Hemoglobin, g/dL	Monopolar TURP	12.39±0.94	11.68±0.93	-0.71
	Bipolar TURP	12.27±1.19	11.69±1.02	-0.58
PCV, %	Monopolar TURP	36.18±2.69	34.44±2.66	-1.74
	Bipolar TURP	35.98±3.08	34.12±3.33	-1.86
Serum sodium, mEq/L	Monopolar TURP	139.46±3.02	133.06±3.16	-6.40
	Bipolar TURP	139.58±3.16	135.18±3.32	-4.40

Postoperative complications

Clot retention occurred in 3 (6%) patients in the monopolar group and 1 (2%) patient in the bipolar group. All cases were treated by clot evacuation. Transurethral resection syndrome developed in 2 (4%) patients in the monopolar group, whereas 0 (0%) patients in the bipolar group developed this complication (Figure 5). The affected patients received intravenous fluids and 3% hypertonic saline.

**Figure 5:**
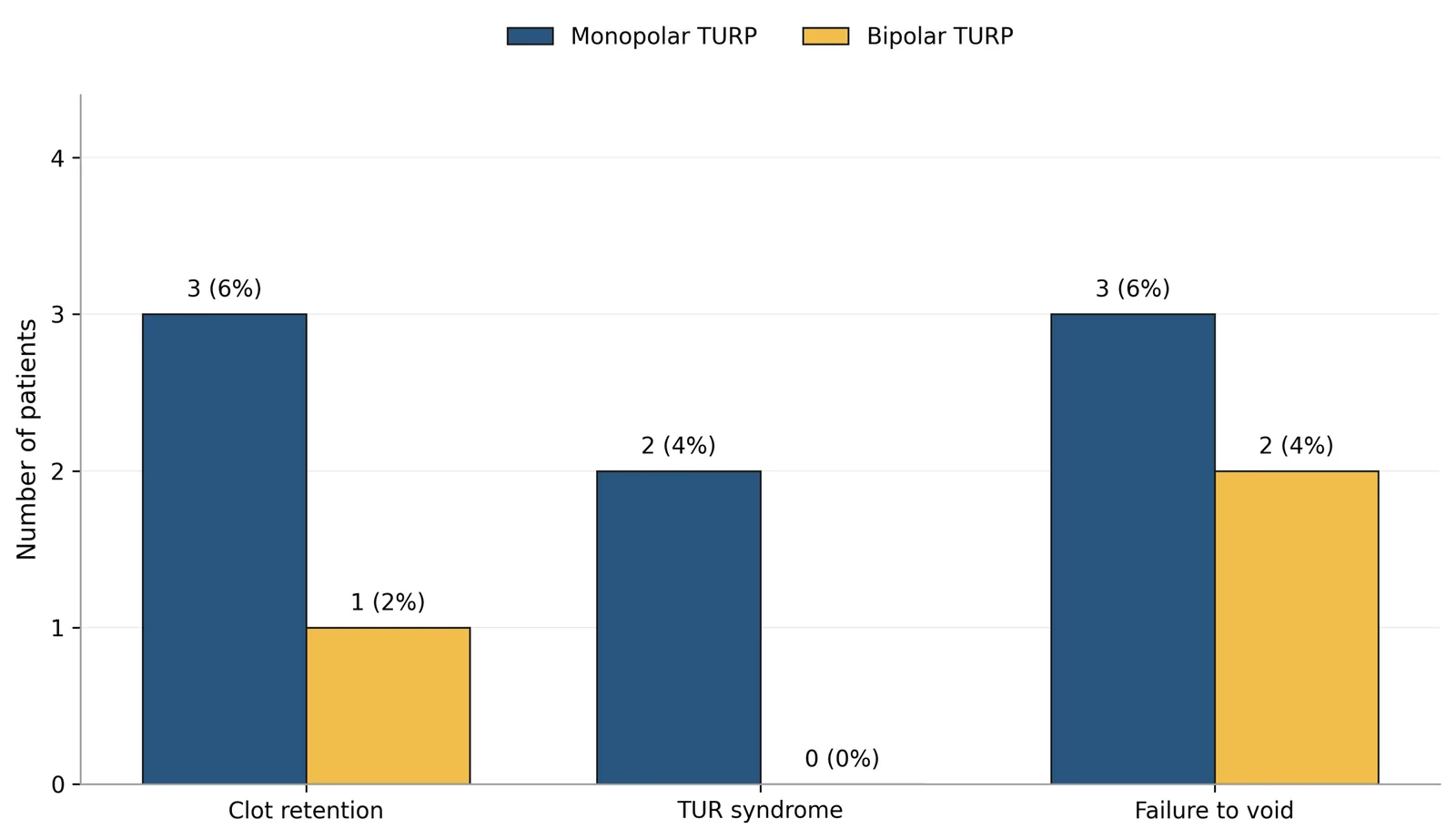
Distribution of postoperative complications TURP: transurethral resection of the prostate, TUR syndrome: transurethral resection syndrome, %: percentage

All urinary catheters were removed on the fourth postoperative day according to the study protocol. Failure to void after catheter removal occurred in 3 (6%) patients in the monopolar group and 2 (4%) patients in the bipolar group (Figure 5). These patients were recatheterized. Table 7 details the early postoperative complications observed in each treatment group.

**Table 7:** Postoperative complications TURP: transurethral resection of the prostate, n: number of patients, %: percentage

Complication	Monopolar TURP (n=50), n (%)	Bipolar TURP (n=50), n (%)
Clot retention	3 (6%)	1 (2%)
Transurethral resection syndrome	2 (4%)	0 (0%)
Failure to void	3 (6%)	2 (4%)

## Discussion

The findings of this study suggest that both monopolar and bipolar TURP are effective in providing significant and similar short-term benefits in urinary symptoms, maximum urinary flow rate, and quality of life in men with benign prostatic enlargement. The techniques, on the other hand, varied in terms of the length of the operation, the level of serum sodium at the end of the surgery, and the observed distribution of early complications. These differences are biologically plausible because monopolar and bipolar electrosurgical systems differ in the direction of current flow, the way in which current interacts with tissue, and the irrigation requirements [16]. The monopolar resection involves non-conductive irrigation and allows the completion of the electrical circuit through the patient, while the bipolar technology localizes the current in the operative field and allows the use of isotonic saline [16].

The mean resection time was significantly longer in the bipolar group compared with the monopolar group. Operative duration is dependent on the surgeon's experience, the equipment type, the tissue response, the prostate configuration, and institutional practice, in addition to the energy platform type [17]. Surgeon preference and technical expertise may also influence the operative performance of monopolar and bipolar systems in routine practice [17]. Therefore, the observed difference in operative time should be interpreted cautiously, as it may also reflect surgeon experience, equipment characteristics, and procedural variability.

Both groups demonstrated substantial improvement in Qmax, IPSS, and quality of life scores after one month. The monopolar group had a slightly higher baseline IPSS than the bipolar group, and this difference was statistically significant (p=0.044). Accordingly, comparisons of postoperative symptom scores should be interpreted cautiously, although both groups demonstrated substantial improvement from their respective baseline values. Between-group comparisons of the postoperative Qmax, IPSS, and QoL values should be reported with their corresponding p-values to support interpretation of comparative functional outcomes. This is consistent with prospective, randomized studies demonstrating comparable urinary flow, symptom, and patient-reported outcome benefits from conventional monopolar TURP and bipolar TURP in saline [18]. Randomized comparisons have similarly shown no significant short-term functional differences, indicating that both methods can successfully relieve obstruction when performed by experienced surgeons [19]. Similar one-year results have been reported elsewhere and support the efficacy of both modalities; however, the present study's one-month follow-up does not allow assessment of durability, reoperation, urethral stricture, or bladder neck contracture [20].

Hemoglobin and packed cell volume were also slightly and similarly reduced in both groups, with no significant difference between groups becoming apparent after surgery. Both methods have previously been described to achieve effective tissue resection with good results, and bipolar systems have been reported to have improved coagulation and reduced bleeding-related morbidity in certain situations [21]. Clot retention occurred in 3 (6%) patients after monopolar TURP and 1 (2%) patient after bipolar TURP. Because no inferential comparison was performed and the number of events was small, this observed difference should be interpreted cautiously. The most significant biochemical change was the more significant decrease in serum sodium following monopolar TURP. Monopolar resection may lead to neurological and cardiovascular symptoms of transurethral resection syndrome, as a result of the fluid absorbed during monopolar resection being hypotonic [22]. Transurethral resection syndrome occurred in 2 (4%) patients in the monopolar group and 0 (0%) patients in the bipolar group. Although the number of events was small, this finding is consistent with the physiological advantage of saline irrigation; however, it does not establish the superiority of bipolar TURP.

Failure to void after catheter removal occurred in 3 (6%) patients in the monopolar group and 2 (4%) patients in the bipolar group. The completeness of the resection is not the only determinant of postoperative voiding status, as other factors such as detrusor contractility, duration of preoperative obstruction, baseline bladder function, and postoperative residual urine dynamics may also play a role [23]. Thus, the observed difference cannot be attributed to the energy system alone. Other endoscopic technologies, such as laser-based procedures, also provide effective relief of bladder outlet obstruction secondary to benign prostatic enlargement, although operative efficiency, bleeding, catheterization, and recovery may differ [24]. In patients with substantially larger prostates, bipolar enucleation may permit more complete adenoma removal than conventional bipolar resection [25]. However, the present findings are most applicable to patients with relatively small prostate volumes.

Strengths, limitations, and future directions

The strengths of this study include its prospective comparative design, direct evaluation of two commonly used TURP techniques, clearly defined eligibility criteria, standardized operative assessment, and evaluation of both functional and perioperative safety outcomes.

There are a few limitations to this study. The non-randomized allocation of surgical technique may have introduced selection bias because treatment assignment could have been influenced by clinical judgment and procedural availability. Although the baseline characteristics were broadly comparable, residual confounding between the groups cannot be excluded. The statistically significant baseline difference in IPSS should also be considered when interpreting postoperative symptom outcomes, as no adjustment for this baseline imbalance was performed. The single-center design also limits generalizability to other centers and patient populations. The relatively small sample size and limited number of postoperative complications reduced the ability to detect statistically significant differences between procedures. Preoperative Qmax data were unavailable for some patients; therefore, analyses involving baseline Qmax were based on the available measurements. Follow-up was limited to one month, and long-term durability of symptom improvement, recurrence of obstruction, urethral stricture, bladder neck contracture, sexual outcomes, reintervention, and delayed complications were not evaluated. Additionally, surgeon expertise and equipment-related factors may have contributed to differences in operative time and perioperative outcomes.

Additional studies should include adequately powered, multicenter randomized controlled trials using standardized surgical techniques, perioperative protocols, and outcome definitions. Follow-up should continue through 12-24 months and involve repeat measures of IPSS, Qmax, quality of life, PVR volume, sexual function, duration of catheter use, hospital stay, and reoperation. Stratified analyses based on prostate volume, baseline urinary retention, comorbidities, and surgeon experience may further improve clinical applicability. Future studies should also evaluate cost-effectiveness, patient-reported satisfaction, and comparisons with laser enucleation and other contemporary endoscopic procedures.

## Conclusions

Both monopolar and bipolar TURP were associated with substantial short-term improvements in urinary symptoms, maximum urinary flow rate, and quality of life in men with benign prostatic enlargement. Both techniques achieved significant improvements in IPSS, QoL, and Qmax at one month. Bipolar TURP had a modestly longer resection time but was associated with a smaller postoperative decrease in serum sodium. Transurethral resection syndrome occurred in 2 (4%) patients in the monopolar group and 0 (0%) patients in the bipolar group, suggesting a potential fluid- and electrolyte-related safety advantage with bipolar TURP in this sample. Hemoglobin and packed cell volume decreased modestly in both groups. Clot retention occurred in 3 (6%) patients in the monopolar group and 1 (2%) patient in the bipolar group, while failure to void occurred in 3 (6%) patients and 2 (4%) patients, respectively. Overall, the findings suggest broadly comparable short-term functional efficacy between the two techniques, with a potentially more favorable fluid- and electrolyte-related safety profile for bipolar TURP. These findings should be interpreted cautiously because of the non-randomized, single-center study design, relatively small sample size, and short follow-up duration. Larger multicenter studies with longer follow-up are required to confirm these findings and assess their durability, complication and reintervention rates, and cost-effectiveness.
